# ERP correlates of German Sign Language processing in deaf native signers

**DOI:** 10.1186/1471-2202-15-62

**Published:** 2014-05-10

**Authors:** Barbara Hänel-Faulhaber, Nils Skotara, Monique Kügow, Uta Salden, Davide Bottari, Brigitte Röder

**Affiliations:** 1 University of Hamburg, Biological Psychology and Neuropsychology, Von-Melle-Park 11, 20146 Hamburg, Germany; 2 Rhine-Waal-University of Applied Sciences, Early Childhood Education, Marie-Curie-Str.1, 47533 Kleve, Germany

**Keywords:** Sign language, Deaf, Native signers, Event-related potentials, Verb agreement, Semantic violation, N400, LAN, P600

## Abstract

**Background:**

The present study investigated the neural correlates of sign language processing of Deaf people who had learned German Sign Language (Deutsche Gebärdensprache, DGS) from their Deaf parents as their first language. Correct and incorrect signed sentences were presented sign by sign on a computer screen. At the end of each sentence the participants had to judge whether or not the sentence was an appropriate DGS sentence. Two types of violations were introduced: (1) semantically incorrect sentences containing a selectional restriction violation (implausible object); (2) morphosyntactically incorrect sentences containing a verb that was incorrectly inflected (i.e., incorrect direction of movement). Event-related brain potentials (ERPs) were recorded from 74 scalp electrodes.

**Results:**

Semantic violations (implausible signs) elicited an N400 effect followed by a positivity. Sentences with a morphosyntactic violation (verb agreement violation) elicited a negativity followed by a broad centro-parietal positivity.

**Conclusions:**

ERP correlates of semantic and morphosyntactic aspects of DGS clearly differed from each other and showed a number of similarities with those observed in other signed and oral languages. These data suggest a similar functional organization of signed and oral languages despite the visual-spacial modality of sign language.

## Background

Sign languages exhibit all characteristics of natural language systems: They have a complex compositional structure, in which signs (analogous to words) are combined to create higher level structures (such as sentences) [1] respectively, [2,3]. However, sign languages remarkably differ from spoken languages with respect to the manner in which they express grammatical relations. Signs are articulated in space and are specified at locations around the signer. This process is called “spatial mapping”. A noun can be specified in the signing space in three different ways: a) by signing the noun and indexing it to a location in the signing space, e.g., the right side of the signer, b) by signing the noun directly at a specific location, or c) by signing the noun and locating it through the starting point of the following verb movement [1]. To express verb agreement, the verb movement and/or palm orientation starts at the location of the subject and ends at the location of the object. Cross-linguistically, sign languages exhibit a strong typological homogeneity in their agreement system [1,4]. This might be due to the fact that all known sign languages use the space around the signer (including the signer). Verb agreement in all known sign languages makes use of these loci in space as well [5]. Despite the linguistic similarities between signed and spoken languages, the surface structure is radically different, such as to use visual-spatial contrasts (i.e., space) to mark grammatical relations in signed languages. Such differences in the surface structure between languages of different modalities offer a unique opportunity to investigate the neurobiology of human language. Native signers are often Deaf individuals born to Deaf parents. Thus, these individuals commonly acquire a sign language from their parents and siblings from an early age. It has been shown that the developmental milestones of natural signed language acquisition correspond to those in natural spoken languages [6,7]. Moreover, lesion and neuroimaging studies on sign language users have suggested a considerable overlap in the neural organization for spoken and sign language processing in Deaf native signers (for a review see [8,9]). As spoken languages, sign languages (like British Sign Language (BSL) and American Sign Language (ASL)) activate the left inferior frontal cortex. Some authors [10,11] have pointed out that the higher activation of homologous right hemispheric structures in sign language processing compared to the processing of a spoken language might be related to the higher reliance of signed languages on spatial functions. However, in these studies ASL processing has been compared with written English. When investigating audio-visual spoken language with sign language no difference in right hemispheric recruitment were observed [12].

To date, relatively little is known about the neural representation of different linguistic domains in sign language processing. Neurolinguistic research of the past years has established reliable event-related potential (ERP) indicators for different aspects of oral language processing [13-15]: Semantic processing (such as lexical expectancy) has commonly been associated with a centro-posterior negativity emerging after e.g., implausible words with a latency of about 400 ms (i.e. the N400 [16-18]). Traditionally, the N400 effect has been assumed to reflect lexical semantic integration processes [19,20].

In contrast, morphosyntactic violations in e.g., verb agreement are associated with a frontal negativity emerging with a latency of approximately 300 ms (the so called LAN [21-24]). This negativity is commonly followed by a positive wave with an onset latency of at least 500 ms (the so called P600 [25] or “syntactic positive shift” (SPS) [13]). This biphasic pattern of a LAN and P600 has been observed for verb agreement violations in various languages [22,24,26-29]. Traditionally, it has been proposed that the LAN reflects early (morpho-)syntactic processing [30,31] and/or working memory functions related to complex processing operations [32]. By contrast, the P600 has been suggested to be associated with processes of syntactic and semantic reanalysis and integration [25,33-35]. However, some researchers have demonstrated that the N400 might be modulated by syntactic processing aspects (e.g., [36]) and the P600 might be modulated by semantic processing aspects under some specific conditions [37,38]. We employed only types of violations for which previous research has demonstrated clearly distinct ERP patterns, that is, an N400 effect for the semantic manipulation and a LAN followed by a P600 for the syntactic manipulation.

In their pioneering studies on the neural correlates of sign language processing, Neville et al. [3,39] compared ERPs to open vs. closed class language elements and reported similar ERP correlates for single signs of ASL and oral word processing. A recent ERP study by Capek et al. has, in addition, investigated the processing of continuously presented ASL sentences [40]. Deaf native users of ASL watched signed sentences that were correct or comprised either a semantic violation (implausible sign) or a morphosyntactic verb agreement error. The Deaf participants, who were all native signers of ASL, showed an N400 effect for semantically implausible signs similar to previously observed effects for corresponding violations in oral languages. By contrast, morphosyntactic verb agreement errors a left frontal negativity followed by a posterior positivity (reversed verb agreement violations with a movement from the object to the subject instead of visa versa) [26,40]. Recently, Hosemann et al. published an N400 effect to unexpected vs. expected sentence final (either action or non-action) verbs of DGS [41].

While the neural correlates of language have been compared between a number of different oral languages, electrophysiological studies on sign languages investigating different linguistic domains to date have mostly concentrated on ASL. To identify the functional organization of sign language comprehension, distinct patterns of neural activation for different linguistic aspects – such as semantic and syntactic processes within the same participants – have to be demonstrated in more than one sign language. The present study employed naturally signed DGS with both semantic (implausible words) and morphosyntactic (verb agreement error, see below) violations. In sum, the present study aimed at determining whether semantic and morphosyntactic aspects of DGS can be dissociated within the same individuals.

Native signers watched continuous DGS sentences, which were either correct or incorrect. Incorrect sentences comprised either an implausible sign or a verb-agreement violation in the middle position of a sentence. We used a different type of verb agreement violation than Capek et al. that can be clearly classified as a morphosyntactic error by sign language linguistics [1]. Moreover, in contrast to Hosemann et al. [41], we introduced the violation in the sentence middle position, because the processing of sentence final words activates additional processes, e.g., related to integration [42]. The participants’ task was to indicate at the end of each sentence whether or not the sentence was correct. In contrast to the stimuli used by Capek et al., the native signer, who signed the sentences was instructed to minimize affective and paralinguistic facial expressions and body movements. This allowed us to minimize coarticulation and paralinguistic effects. We expected distinct ERP patterns for semantic and morphosyntactic violations: A centro-posterior N400 like effect was predicted for semantic and a frontally distributed negativity followed by a posterior positivity was predicted for morphosyntactic violations.

## Results

### Behavioral data

The analysis of the percentages of correct responses revealed that Deaf native signers (n = 11) correctly judged 96.71% (SE: 0.58%) of the correct sentences, 98.42% (SE: 0.78%) of semantically incorrect sentences and 96.45% (SE: 0.12%) of morphosyntactically anomalous sentences.

### EEG data

#### Semantic condition

**Figure 1:**
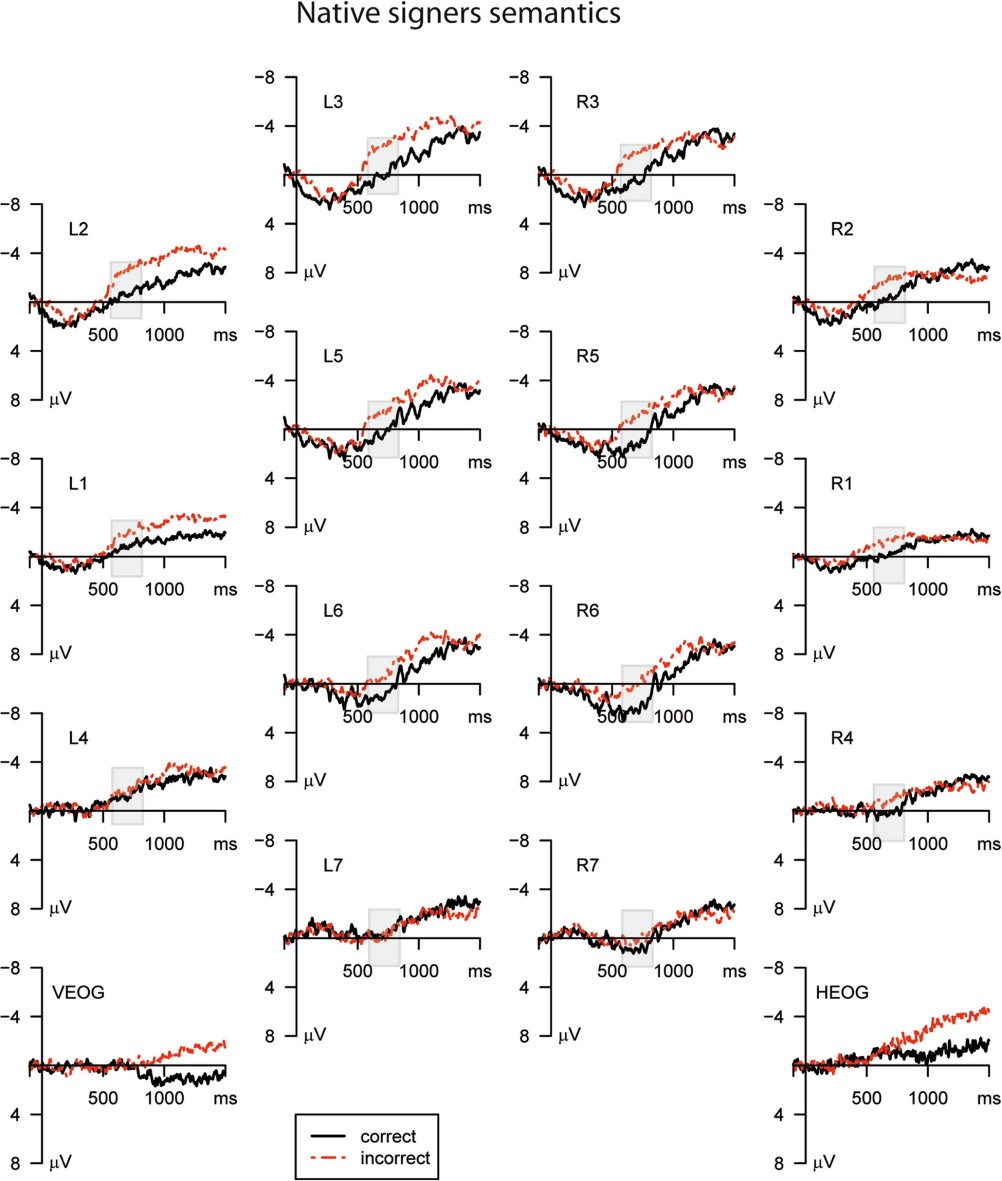
**Mean ERPs semantic condition.** Mean ERPs in the semantic condition for the Deaf native signers for all clusters. The dotted lines denote the ERP in the incorrect condition; the solid lines denote the ERP in the correct condition. The analyzed time epoch is marked with a grey box.

The ERPs of Deaf native signers for the critical verb of correct sentences and semantically incorrect sentences are displayed in Figure 1. Semantically incorrect sentences elicited a more negative going potential compared to the correct sentences. This observation was confirmed by a main effect of Condition (F(1,10) = 6.267; p = 0.031) for the time window of **550–750 ms**. Furthermore, a significant interaction of Condition and Cluster (F(2.8,28.1) = 3.253; p = 0.039) was revealed indicating a bilateral fronto-central scalp distribution of the violation effect. The latter was significant at clusters L1, L2, L3, L5, L6, R1, R2, R3, R5, and R6 (p < 0.05).

#### Syntactic condition

**Figure 2:**
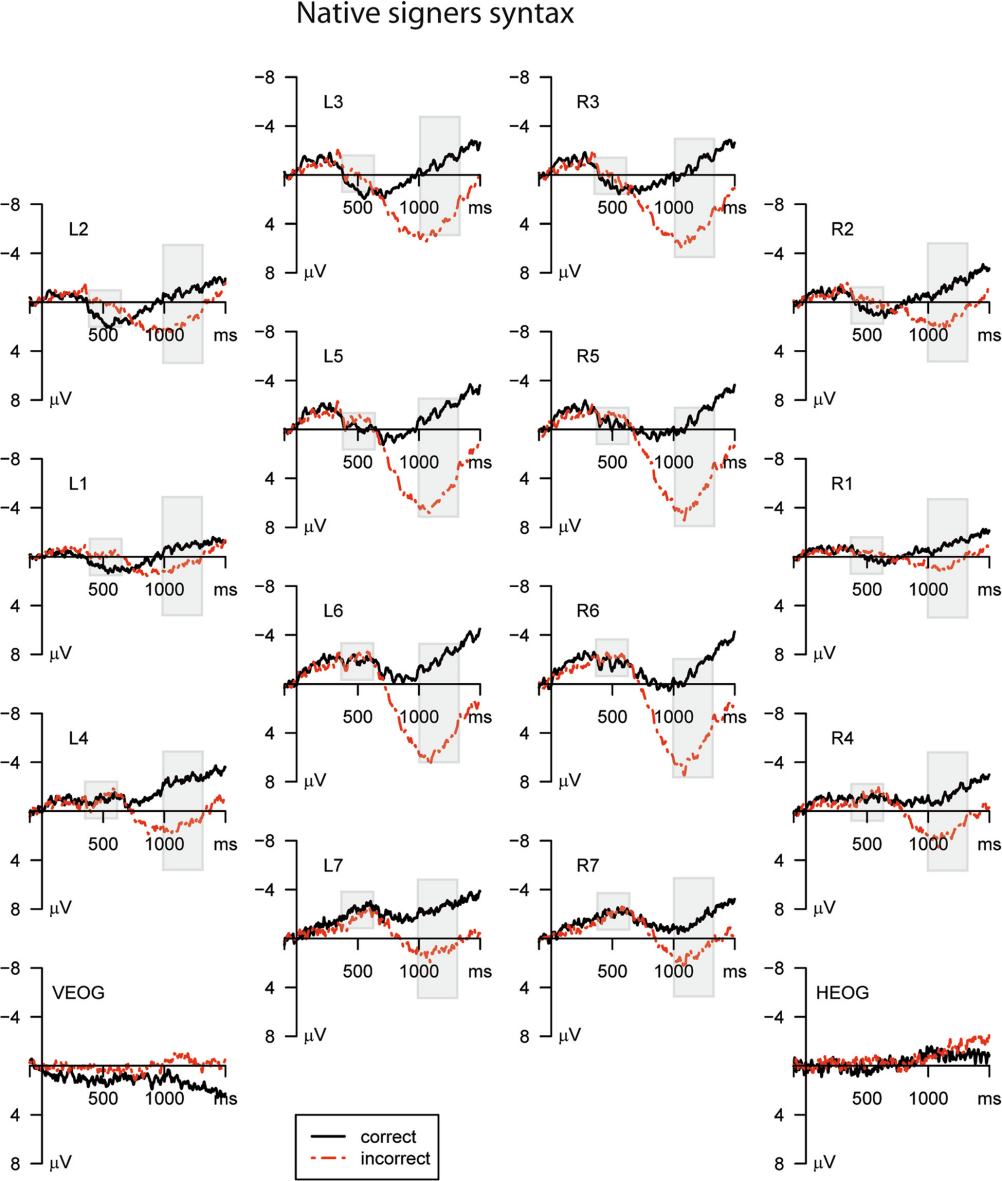
**Mean ERPs syntactic condition.** Mean ERPs in the morphosyntactic condition for the Deaf native signers for all clusters. The dotted lines denote the ERP in the incorrect condition, the solid lines denote the ERP in the correct condition. The analyzed time epoch is marked with a grey box.

**Figure 3:**
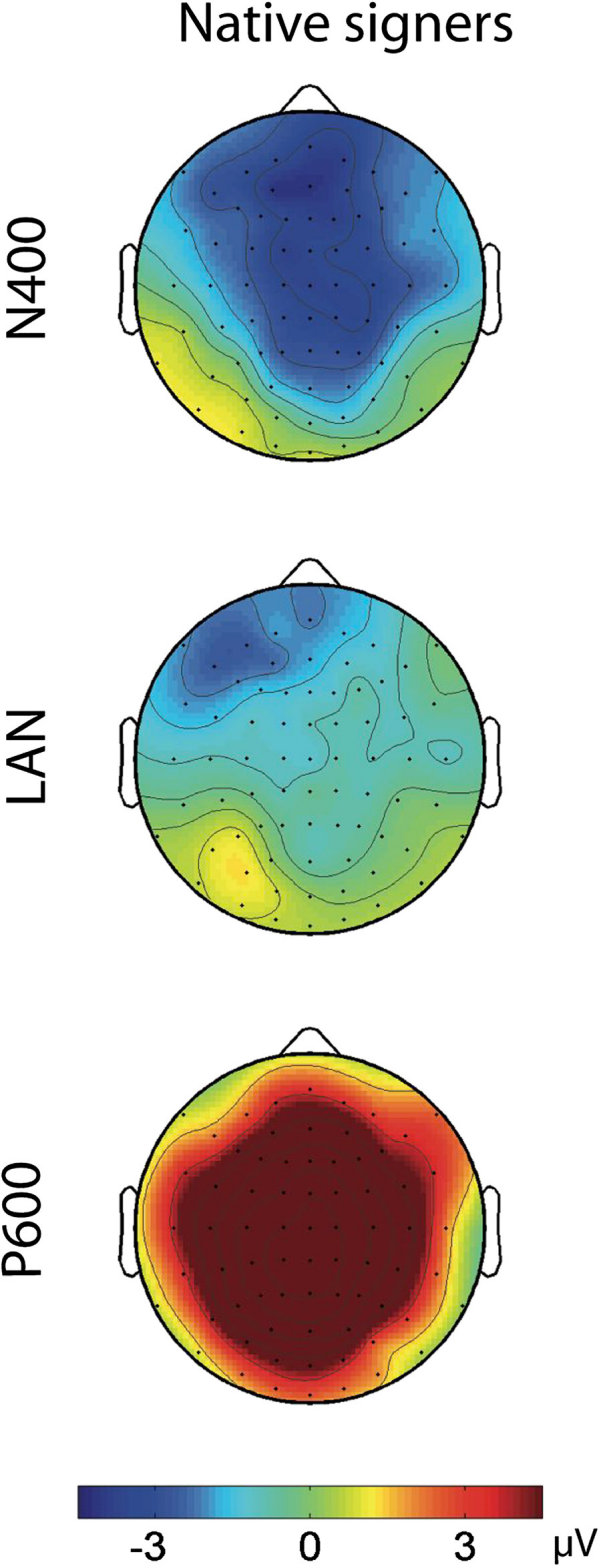
**Overview Topographic Distributions of the ERPs.** Topographies of the N400 (550–750 ms, first row), LAN (400–600 ms, second row), and P600 (1000–1300 ms, third row). Blue denotes negative values and red denotes positive values in μV.

The ERP data for the critical verb for Deaf native signers for correct sentences and morphosyntactically incorrect sentences are shown in Figures 2 and 3. Morphosyntactically incorrect sentences elicited a negative potential (LAN) in the time epoch 400–600 ms and a positive wave (P600) in the time epoch 1000**–**1300 ms.

The ANOVA [43] revealed a three way interaction of Condition and Hemisphere by Cluster (F(2.7,26.6) = 3.991; eps = 0.443; p = 0.021) in the time window **400–600 ms** indicating a typical left lateralized frontal distribution of the LAN. The negative difference between correct and incorrect sentences was significant at clusters L1, L2, L3 (p < 0.05).

A significant main effect of Condition (F(1, 10) = 42.346; p < 0.001) was observed in the second time epoch of **1000–1300 ms,** confirming a larger positivity in the response to morphosyntactic violations compared to morphosyntactically correct signs. In addition, the interaction of Condition by Cluster was significant (F(1.6,16.1) = 13.110; eps = 0.268; p < 0.001), indicating the typical posterior distribution of the P600. The positive difference was significant at all clusters (p < 0.05).

## Discussion

Semantic and morphosyntactic violations addressing two functionally different linguistic aspects within German Sign Language (DGS) elicited clearly distinct ERP patterns. Semantic violations (implausible signs) were followed by a negative ERP with a fronto-central scalp distribution (N400). By contrast, syntactic violations (verb agreement violations in DGS) elicited a frontal negativity (LAN) followed by a central positivity (P600).

The N400 observed in native signers had a more anterior distribution than observed in reading studies [16,44-46]. A more anterior distribution of the N400 has been reported for auditory stimuli as well [47,48], i.e., a modality that resembles sign language in its temporal dynamics more than written language does.

By contrast, other sign language studies have found a more posteriorly distributed N400 effect for both single noun signs and implausible nouns in sign language sentences [3,40]. This effect could be explained by the processing of different word classes. In contrast to ASL, DGS has a different word order: while ASL is a subject-verb-object language, DGS belongs to the subject-object-verb languages. Therefore, the semantic violation is only detectable at the verb that follows the implausible object. Hence, the critical sign in the present DGS study was the verb rather than the object, as it was the case in the study of Capek et al. [40].

Several studies have shown that processing words from different grammatical classes (nouns and object words vs. verbs and action words, open vs. closed class, nouns vs. verbs) seem to engage different neural networks [49,50]. Thus, another account for the different distributions of the N400 effect in the present and in the study of Capek et al. [40] may be the use of critical words from different word classes. Indeed, in an accompanying experiment with written German sentences, we found a similar posterior topography of the N400 in the Deaf native signers as observed in hearing L1 and hearing L2 users of German [51]. In this study the critical word was the object.

In a recent ERP study on DGS, Hosemann et al. observed an N400 to action and non-action verbs but did not discuss the topography of the N400 effect for unexpected action vs. non-action words. From their figures it seems as if the N400 effect to the non-action verbs had a more posterior distribution than the N400 effect to the action verbs. Since we used action verbs in our study, this result would fit nicely with our data.

By contrast, morphosyntactic violations elicited a left lateralized negativity with an anterior distribution. The anterior negativity was followed by a broadly distributed positivity with a central maximum. Both effects were highly similar to what has been observed previously, following both a large number of different syntactic anomalies in aural-oral languages [21,25] and verb agreement violations in ASL [40]. However, our effects emerged later than in these studies [40]. These latency differences can be explained by the different trigger positions for ERP timelocking. As mentioned in the methods sign languages have long transition phases between two consecutive signs. Since in the morphosyntactic condition the location change of the sign is more crucial than the target sign itself we set the trigger position to the first detectable change in location with respect to the preceeding sign. This early trigger position caused the late onset of the violation effects. Indeed, Hosemann et al. showed for their N400 effects that changing the trigger along the transition phase changes the timing of the N400 effect.

Particularly impressive in our data is the clear left lateralization of the anterior negativity (LAN effect) which has previously been reported as a response to verb-agreement violations both in spoken English and German [24,26,35]. Our results extend the findings of Capek et al. [40]. Remarkably, the verb agreement violation used in our study differed from the two used by in this study: As a verb agreement violation they used reversed movement from the object location to the subject location instead of visa versa or a movement from the correct subject location to a non-defined location in space. Capek et al. suggested that the larger right hemispheric distribution of the violation effect for the second compared to the first violation type was mainly due to the increased spatial mapping requirements for non-localized signs in space. In contrast, in our study we combined the use of an unspecified and a wrong location in one verb agreement violation: the verb moved from a neutral (unspecified) subject position to the deictic first person object location. Compared to Capek et al., we clearly introduced the referential positions of subject and object in the signing space before the verb sign making spatial mapping by verb movement unlikely. Though in our paradigm the verb movement starts at an unspecified location as well, the starting point of the verb movement does not allow for a localization of the subject, because it has already been located at a different place before. Thus, the verb movement was clearly grammatically incorrect. Hence, no additional mapping process was necessary. The error between referential positions and verb movement was **–** as in typical syntactic parcing processes **–** most likely to be automatically detected as a morphosyntactic mismatch. This is explaining why we found a clearly left lateralized anterior negativity, similar to that observed in the ASL reversed verb agreement condition of Capek et al.

## Conclusions

Consistent with previous research on oral and signed languages, we provide evidence that semantic and syntactic aspects of DGS are distinct processes, i.e., processes mediated by different neural systemes.

## Methods

### Participants

Fifteen Deaf native signers (≥85db Hearing level (HL) in each ear except for one participant who had a decibel loss of ≥ 70 db HL in the left ear) participated in the experiment. Three participants had to be excluded from further analyses since they did not reach the criterion of at least 60% correct responses in all experimental conditions. Additionally, one participant was excluded because the EEG data set was contaminated by excessive artifacts. Of the analyzed sample (6 female, 5 male; mean age: 28 years, range: 20**–**40 years), four participants had “mittlere Reife” (correspondent approximately to an O-level), seven had “Abitur” (A-level), and one had an university degree. The first three excluded participants had “mittlere Reife”, the fourth excluded participant did not report his highest degree of education.

None of the participants had any known neurological impairments and all of them had normal or corrected-to-normal vision. They gave written informed consent before their participation and received a monetary compensation. All of the native signers were right-handed according to self-report and the Edinburgh Handedness Inventory. The participants had learned DGS from birth from their Deaf parents.

The sign language proficiency of the participants was assessed by using a DGS comprehension test (Gebärdensprach-Sinnverständnis Test (GSV) of the ATBG (“Aachener Testverfahren zur Berufseignung von Gehörlosen”; English: “Aachen’s vocational testing for the deaf”). On average, the selected participants were 87% (SE 3.7, range: 60% to 100%) correct in the DGS comprehension test. The study had been approved by the ethical committee of the German Society of Psychology (Nr: BRBHF 07022008).

### Material

A set of 300 experimental sentences was constructed by two Deaf native signers, one Deaf near-native signer of DGS, and one sign language linguist. The sentences were signed by a Deaf native signer of DGS, videotyped, digitized, and presented at the rate of natural signing. Written informed consent for the publication of images was obtained from the signer.

The stimulus set was evaluated by 12 congenitally and profoundly deaf individuals (mean age: 36 years, range: 27**–**64 years; ≥ 85db HL in each ear) who were all native signers of DGS. Upon presentation, participants had to judge whether or not the sentence was an appropriate DGS sentence. Sentences with less than 80% agreement among the native signers were disregarded. The final stimulus set consisted of 46 sentences from which 138 sentences were derived: (a) 46 sentences were correct, (b) 46 sentences were morphosyntactically incorrect comprising a verb that was incorrectly inflected (incorrect direction of movement), and (c) 46 sentences were semantically incorrect comprising a selectional restriction violation. For example, sentence (1b) violates the person agreement rule between subject verb and object verb via the wrong movement from neutral space to the first person:

(1a) BOY POINTa GIRL POINTb *aNEEDLEb* REASON POINTb SLOW SWIM

“the boy needles the girl because she is slowly swimming”

(1b) * BOY POINTa GIRL POINTb *cNEEDLE1* REASON POINTb SLOW SWIM

“*the boy needle the girl because she is slowly swimming”

Instead, sentence (1c) is an example of a selectional restriction violation (semantic violation), since the object-verb relation is not semantically plausible:

(1c) BOY POINTa *COAT* POINTb aNEEDLEb REASON POINTb SLOW SWIM

“*the boy needles the coat because it is slowly swimming”

**Figure 4:**
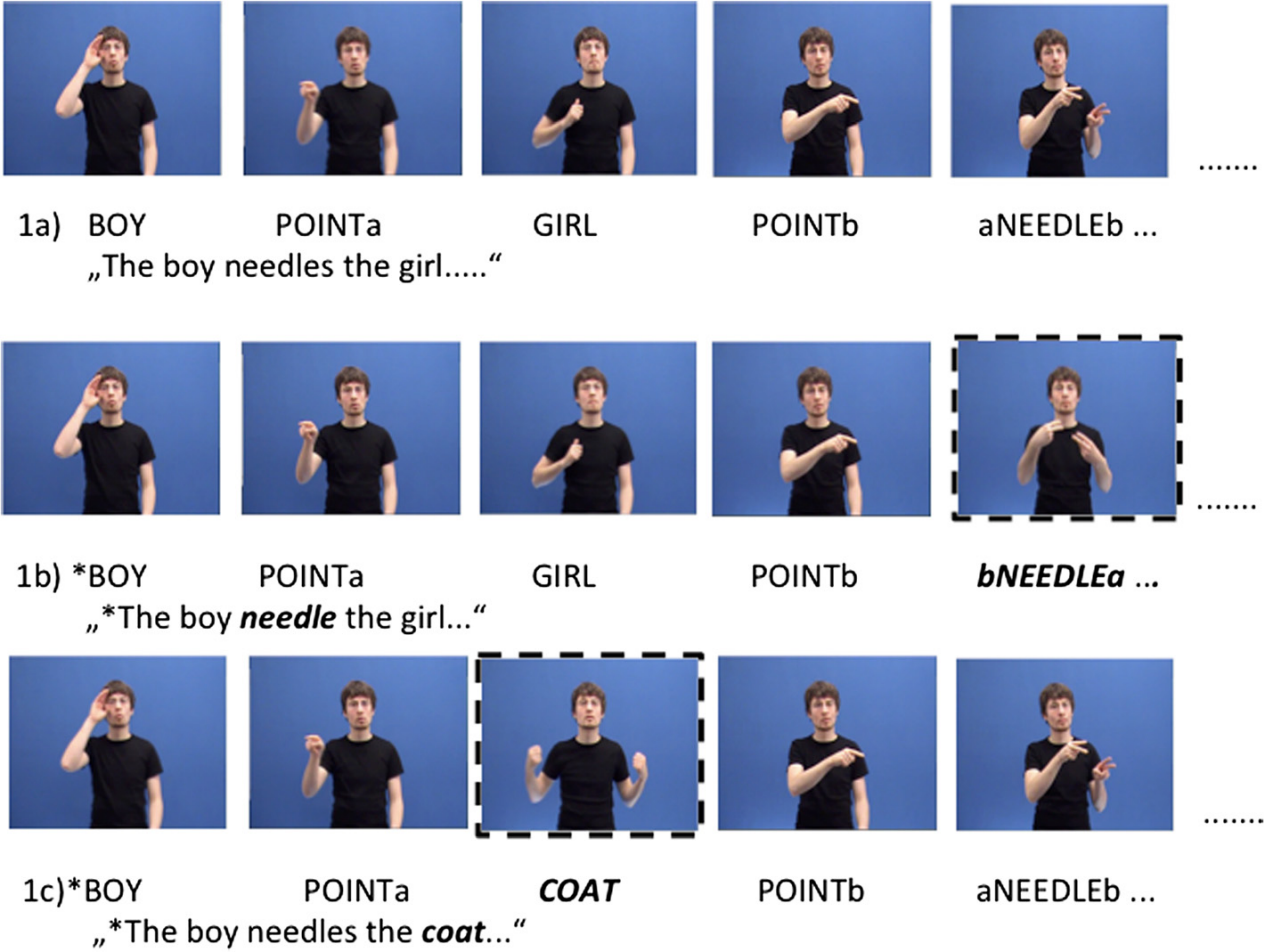
**Still image samples illustrating the two types of violations in the DGS sentences.** Notations in lowercase indicate the location of the sign: a = right of the signer; b = left of the signer; c = in front of the signer; 1 = signer; POINT = pointing to a location to place the referent in the signing space; the errors are marked through dotted lines resp. bold prints **1a)** correct sentence: the verb sign NEEDLE moves correctly from the subject location to the object location; **1b)** morphosyntactically incorrect sentence: the verb sign NEEDLE moves incorrectly from an unspecified location in front of the signer to the signer indicating first person; **1c)** semantically incorrect sentence: semantically implausible object sign COAT.

All sentences were constructed in a comparable SOV structure up to the critical sign (Figure 4).

Since DGS is a subject-object-verb (SOV) language, the semantically violated sentences became implausible at the verb (e.g., NEEDLE in the example shown in 1c). Thus, the verb is the critical sign to which ERPs were averaged.

Sentences had a mean length of 10 signs (median: 9, range: 7 – 13 signs) and a mean duration of 10457 ms (median: 10440 ms, range: 5680 ms–14480 ms, SD 1596). Additionally, 74 different filler sentences were presented. Sixty filler sentences were correct, 14 sentences had different morphosyntactic and semantic violations on varying sentence positions.

The stimulus onset of each sign was defined by a Deaf native signer, a Deaf delayed signer, and a DGS interpreter. Sign languages have rather long transition phases between one sign and the next [52] and it is a matter of debate when exactly a sign starts. According to Liddell & Johnson a sign begins when the handshape is completed and the hand is hold in its correct first location (‘Movement-Hold-Model’; [53]). Note, however, that the timing of comprehending a sign varies depending a) of what signing parameters has to be changed and b) of which signing parameter is linguistically crucial. Therefore, in our paradigm we distinguished two time points which were used as trigger positions (event codes):

1. In sentences with semantic violations we timelocked the sign onset – according to the Movement-Hold-Model – when handshape and hold were completed: To judge the semantic value of the object the target sign has to be perceived entirely in order to judge its appropriateness.

2. In sentences with morphosyntactic violations the location change (note that in sign language, syntax is expressed in space) of the sign is more crucial than the target sign itself: while moving the hand to the location of the beginning of the next sign the handshape changes and the morphosyntactic violation (incorrect location) is most likely recognized. Therefore, for morphosyntactically violated sentences the trigger position was set to the first handshape change that could be detected towards the target sign or – if earlier – the change of the lip movement (sign onset code II).

### Procedure

The experiment comprised two sessions that were run mostly within one day. In the first session, the participants completed a language history questionnaire and a subtest of the ATBG (“Aachener Testverfahren zur Berufseignung von Gehörlosen”; English: “Aachen’s vocational testing for the deaf”). The test comprises a number of different modules to test aspects of memory, attention, spatial imagery, problem solving, general knowledge, arithmetic, and language. We only employed the subtest GSV (“Gebärdensprach-Verständnis-Test”; English: “Sign Language comprehension test”).

The experimental session consisted of 212 trials and was divided into five blocks with short breaks of a duration defined by the participants. The experiment lasted for about 90 minutes. Prior to the experimental blocks, 13 practice sentences were presented (which were not used in the analysis). Instructions were given in DGS: Since signers are familiar with a wide range of variations in DGS within the German signing community, they are extremely tolerant for language variation. For this reason participants were told to only accept “very well-formed” sentences as “correct”.

Participants were seated in a comfortable chair in front of a LCD monitor. Stimuli were presented on this monitor with a vertical visual angle of 13.12° and a horizontal visual angle of 16.48°. The size of the presented video footage was chosen to be readily identifiable.

Please note that the visual angels refer to the complete size of the shown footage. Thus, the visual angles within which the relevant signing was presented were smaller. In addition, during sign language comprehension, signers fixate primarily on the signers face (see results from eye tracking studies e.g., [54,55]).

The different trial types were presented in a random order, holding the first picture of the video/signed sentence for 1000 ms with the signer in initial position to fixate the participant’s eyes on the screen. Six hundred ms after the end of the sentence a happy and a sad smiley appeared on the screen and participants were prompted to decide whether or not the sentence had been correct by pressing one of two buttons with their left and right index fingers (which hand was used to indicate correct and incorrect sentences, respectively, was randomized across participants). To start the next trial, the participants had to press one of the response buttons. In the second session, participants’ processing of written German sentences was examined (see [51], [56]).

### ERP recording and data analysis

The electroencephalogram (EEG) and the electro-oculogram (EOG) were recorded using Ag/AgCl electrodes. Seventy-four electrodes were mounted according to the international 10/10 system into an elastic cap (Easy Cap; FMS, Herrsching-Breitbrunn, Germany) (see Figure 2). The vertical EOG (VEOG) was recorded with an electrode below both eyes against the right earlobe reference. The horizontal eye movements were monitored using electrodes F9 and F10 (bipolar recording defined offline). An averaged right/left earlobe reference was calculated offline. Electrode impedance was kept below 5 kΩ. The electrode signals were amplified using 3 BrainAmp DC amplifiers (Brain Products GmbH, Gilching, Germany) and digitally stored using the BrainVision Recorder software (Brain Products GmbH, Gilching, Germany). The analog EEG signal was sampled at 5000 Hz, filtered online with a bandpass of 0.1 to 250 Hz and then downsampled online to 500 Hz to be stored on a disk. The signal was low pass filtered offline with a high cut-off at 40Hz, 12 dB/oct.

**Figure 5:**
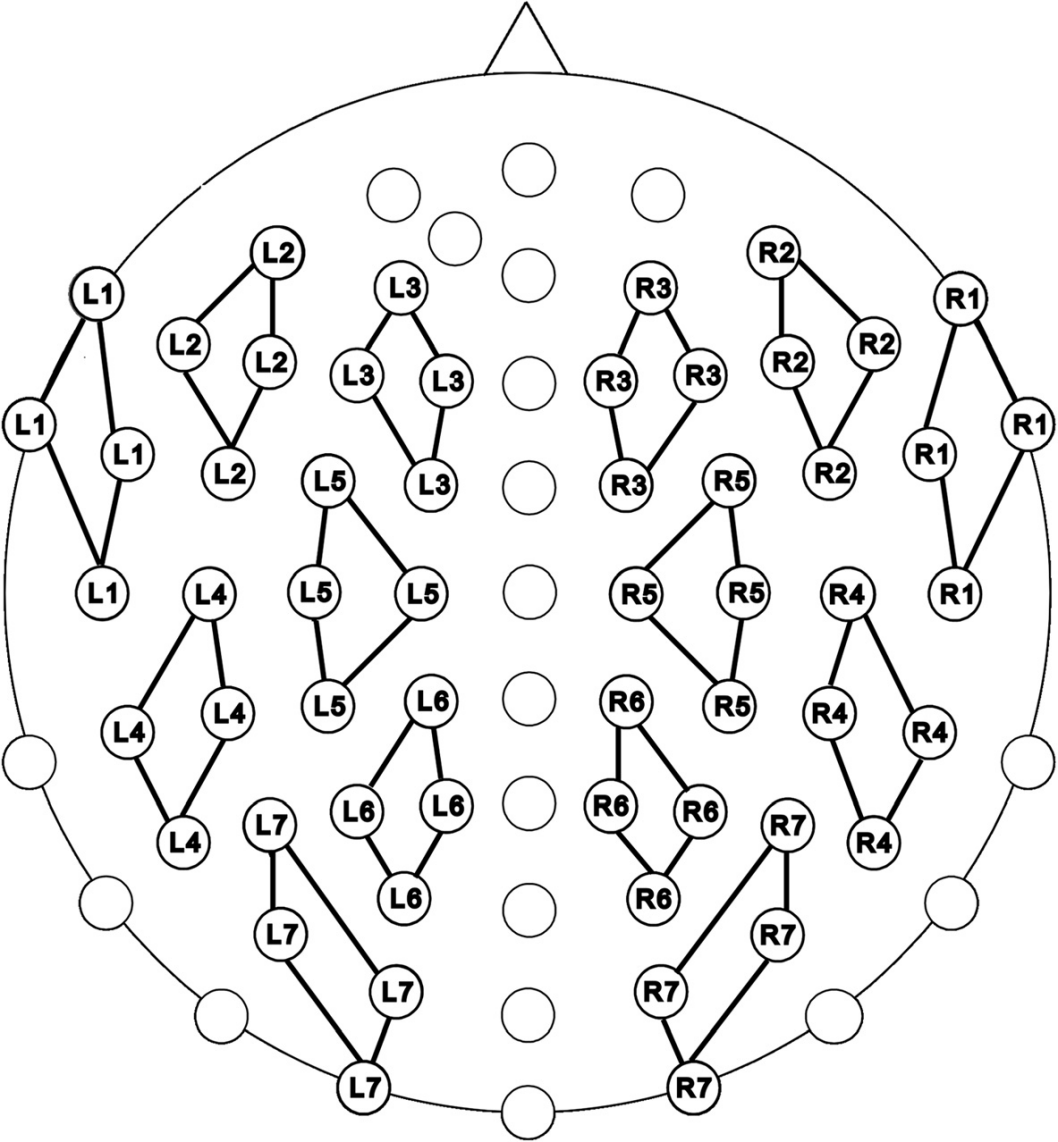
**Schematic illustration of the electrodic clusters.** The 14 clusters, 7 on each hemisphere, were used for the statistical analyses and are marked by connecting lines.

Since language related ERPs have a rather broad topography, four adjacent electrodes were pooled, resulting in 7 electrode clusters for each hemisphere (see Figure 5).

The behavioural data (percentage of correct judgements) were analyzed with a repeated measurements ANOVA with the within participant factor Condition (correct, semantically incorrect, and morphosyntactically incorrect).

Trials with ocular artifacts (with an individually adjusted criterion of a maximum peak to peak amplitude between 80–120 μV within the time epoch of −100–1500 ms), artifacts from muscle movements, alpha waves or drifts,which had an individually adjusted criterion up to 150 μV within the time epoch of −100–1500 ms for each participant, were identified and rejected offline.

The remaining segments were baseline corrected with respect to a 100 ms period preceding the onset of the critical word. Separate averages were calculated for the four conditions (1a) correct (sign onset code I), (2) semantically incorrect (sign onset code I), (1b) correct (sign onset code II), (3) morphosyntactically incorrect (sign onset code II) for the time segment starting 100 ms before and ending 1500 ms after the critical words.

Based on results from running t-tests and a visual inspection of the data, we ran analyses on the mean voltage of the following time epochs: 550**–**750 ms (N400) for semantic violations and 400**–**600 ms (LAN) and 1000**–**1300 ms (P600) for morphosyntactically violated sentences.

Time epochs were separately analyzed with an ANOVA comprising the repeated measurement factors Condition (correct vs. incorrect), Hemisphere (left vs. right), and Cluster (1**–**7). Sums of squares of Type II were calculated. To compensate for violations of the assumption of sphericity in multi-channel electroencephalographic data, the Huynh and Feldt correction was applied. Corrected degrees of freedom and corrected p-values, as well as the Huynh and Feldt epsilons (eps) are reported for the F-tests in the result section. Statistically significant effects without the factor Condition are not reported. The difference of the incorrect and the correct condition was tested with one tailed t-tests at each cluster. To correct for unequal variances, the degrees of freedom of the t-tests were corrected using the Welch algorithm [57]. The open source statistical programming language “R” was used for statistical analyses.

Regarding the participants, only trials followed by a correct response were included in the analysis. If a participant made more than 40% mistakes in at least one condition, he or she was excluded from the analysis. As described in the section participants, three Deaf native signers were excluded due to low performance.

## Competing interests

We hereby declare that we do not have received reimbursements, fees, funding, or salary from an organization that may in any way gain or lose financially from the publication of this manuscript, either now or in the future. No such an organization is financing this manuscript. We do not hold any stocks or shares in an organization that may in any way gain or lose financially from the publication of this manuscript, either now or in the future. We do not hold or are currently applying for any patents relating to the content of the manuscript. We have not received reimbursements, fees, funding, or salary from an organization that holds or has applied for patents relating to the content of the manuscript. We do not have any other financial competing interests. We also declare that there are also no non-financial competing interests (political, personal, religious, ideological, academic, intellectual, commercial) or any other in relation to this manuscript.

## Authors’ contributions

BHF, NS and BR designed the experiment. MK and US run the ERP experiments. NS, DB and BR analyzed the data. BHF, NS, US, DB and BR wrote the paper. All authors read and approved the final manuscript.
